# Design and Prototyping a Novel Hybrid Shoulder Exoskeleton

**DOI:** 10.3390/biomimetics11070442

**Published:** 2026-06-24

**Authors:** Joel Quarnstrom, Abram Smith, Owen Barragan, Adrian Toquothty, Yujiang Xiang

**Affiliations:** School of Mechanical and Aerospace Engineering, Oklahoma State University, Stillwater, OK 74078, USA; joel.quarnstrom@okstate.edu (J.Q.); abram.smith@okstate.edu (A.S.); rody.barragan@okstate.edu (O.B.); adrian.k.toquothty@okstate.edu (A.T.)

**Keywords:** shoulder exoskeleton, hybrid system, mechanism design, helical actuators, computational kinematics

## Abstract

Shoulder injuries due to labor-related lifting tasks are widespread in manufacturing and logistics companies. Prolonged shifts and repetitive motions lead to muscle fatigue, significantly elevating the risk of both acute accidents and chronic musculoskeletal disorders. Many passive exoskeletons which use springs to provide lifting assistance have been commercialized, and many active exoskeletons have been researched. The drawback to passive exoskeletons is the larger the lifting force that they produce, the larger the force required to lower the arms. This contributes to tiring the user. Conversely, active exoskeletons require substantial energy to provide meaningful torque. Furthermore, they pose a safety risk; a sudden power failure could result in an instantaneous loss of support, potentially causing the user to drop a heavy load and sustain injury. This research project proposes a hybrid exoskeleton with a parallel elastic actuator that uses a motorized helical actuator which can be tuned to improve lifting performance. This paper evaluates the kinematics and statics of the proposed exoskeleton, details the design and implementation of the electrical control system, shows mechanism optimization of the mechanical advantage profile, and validates the concept through the construction and experimental testing of a functional prototype.

## 1. Introduction

Work-related shoulder injuries remain a critical concern in industrial environments characterized by manual material handling, maintenance, and construction. In 2024, the National Safety Council (NSC 2024) reported over 34,000 DART (days away, restricted, transferred) shoulder injuries due to either overexertion in lifting or repetitive motion involving microtask events [1]. These occurred in the material handling, production, maintenance, and construction industries. Many of these injuries occurred in warehouse or logistics operations with workers lifting boxes or other heavy objects. Also, shoulder injuries can be caused by over-the-head tasks such as drilling, wiring, and rebar installation. These injuries can be either chronic or acute. Fatigue discomfort can make workers less careful in their actions which increases the chance of workplace injury or accident. One solution to this problem is to provide workers with assistive devices, such as shoulder exoskeletons, that reduce the load on their shoulders to decrease fatigue and decrease injury.

There are many passive exoskeletons on the market that reduce shoulder load during lifting. These include Ottobock’s IX Shoulder Air, Crimson Dynamics’ CDYS, and HILTI’s Exo-O1 and Exo-S [2]. These products use passive spring elements to provide upward lifting force on the user’s upper arm. The biggest drawback to these products is that when the user is not carrying any weight and they need to lower their arms, they have to exert force to work against the spring to lower their arms. This will cause extra wear on the user’s muscles which is not desirable. The technique that these exoskeletons use to combat this problem is to adjust the spring tension so that the arm-lowering force is not too much. This in turn lowers the lifting force which reduces the exoskeleton’s assistive ability.

There are also powered shoulder exoskeletons with motorized actuators including the MAPS-E, EXOIQ S700, and Agadexo Shoulder, which is a hybrid shoulder exoskeleton. Other assistive lifting exoskeletons have support linkages that do not contact the arm at all, and instead directly contact the hand and are integrated into some type of glove or lifting hook [2]. This last style will not be considered in this research, and only shoulder exoskeletons that provide lifting force to the arm will be compared.

The drawback to purely active exoskeletons is that in the case of power failure, if the drive mechanism is not backdrivable, the user could drop a heavy weight and cause accident or injury. Also, for backdrivable mechanisms, the motor will draw a lot of power to constantly maintain a lifting force. For non-backdrivable purely active mechanisms (which were not found on the market), a lack of backdrivablilty would make operating the exoskeleton too rigid and possibly jarring. The Agadexo Shoulder is a series hybrid exoskeleton with a non-backdrivable actuator in series with an elastic component to provide lifting force to the upper arm. The elastic component smooths the rigidness of the non-backdrivable actuator, and it in turn saves actuation power by utilizing the non-backdrivability of the mechanism to maintain stall loads which is clever. The drawback to this mechanism is that the spring provides some latency or attenuation of the control actuation which could cause feedback or precision issues.

Research has been conducted not only on the design of novel shoulder exoskeleton mechanisms but also on their evaluation in both laboratory and field settings. One of the primary challenges in shoulder exoskeleton design is achieving precise alignment between the device’s joints and the user’s anatomical shoulder joint. This difficulty arises in part from the shoulder’s small but significant translational degrees of freedom (DOFs). To address this issue, researchers in [3] employed a rotational series elastic actuator (SEA) to generate shoulder lifting force. The actuator was vertically supported by a “hyper-redundant” passive mechanism, enabling both rotational and translational motion in the transverse plane. Researchers in [4] also treated this problem by adding a three DOF follower mechanism to their active shoulder exoskeleton to accommodate the shoulder translations.

A simulation study in [5] explored a shoulder exoskeleton based on a parallel mechanism driven by two pistons. This design sought to mitigate joint misalignment by incorporating additional passive DOFs and utilizing the piston actuators in a semi-redundant configuration. Notably, this system applies lifting force to the forearm rather than the upper arm. In another approach, researchers in [6] developed a passive shoulder exoskeleton using linear springs and a cam mechanism to generate upward lifting torque. The cam profile could be customized to produce a desired torque–angle relationship. Similarly, a passive exoskeleton in [7] utilized a rotational magnetic spring composed of a permanent magnet stator and rotor, offering reduced friction and a tunable force profile. A string-based rotational spring was also proposed in [8] to provide assistive torque, with adaptability to different users, and a spring-based cable driven passive exoskeleton in [9] allowed for a wide range of shoulder motion while providing lifting force. A passive exoskeleton using an active magnetorheological breaking system in [10] gave users a large amount of joint locking torque to perform carry and hold tasks without using motors. This hybrid system demonstrates low power, high stationary torque, passive exoskeleton system, but does not solve the problem of the user fighting the passive exoskeleton spring when lowering their arms without holding weight. Researchers designed and tested a passive exoskeleton in [11] and used an optimizable linkage mechanism to transmit the lifting torque to the user’s shoulder. An active exoskeleton demonstrated a novel sensor fusion algorithm for active control in [12]. A robust controller is critical to the usefulness of active exoskeletons. Lastly, an exoskeleton kinematic simulation scheme was demonstrated in [13].

In addition to design innovations, several studies have investigated the effectiveness of shoulder exoskeletons in practical applications. Researchers in [14] conducted human trials to evaluate the impact of an active shoulder exoskeleton on overhead drilling tasks, demonstrating a reduction in shoulder strain. A commercial passive exoskeleton, ShoulderX, was evaluated in an industrial setting with 20 healthy adult workers, where 10 participants used the device and 10 served as controls [15]. However, inconsistent usage over the multi-week study led to inconclusive results, highlighting broader ergonomic and adoption challenges. Another study compared ShoulderX with the SuitX passive exoskeleton for industrial lifting tasks and found that both systems provided only limited assistance under the tested conditions [16].

Zaman et al. [17] reviewed optimization-based biomechanical models for lifting task design, simulation, and analyses. Arefeen and Xiang [18] optimized the powered knee exoskeletons to aid human lifting tasks. The optimal control of the exoskeletons were validated with experimental results. In addition, artificial neural network was used to control the powered knee exoskeletons in real time [19].

The existing commercial and academic shoulder exoskeletons use a variety of active and passive mechanisms to provide assistive lifting force. The gap in the state of the art for passive exoskeletons is that they produce antagonistic force for the user to lower their arms whose magnitude is directly correlated with the maximum assistive force. The gaps for active exoskeletons are that they consume large amounts of energy to provide meaningful lifting force for backdrivable mechanisms and do not provide dynamic torque control for non-backdrivable mechanisms.

To bridge these gaps, this work proposes a hybrid shoulder exoskeleton that combines a parallel elastic actuator with a novel motorized helical actuator. This architecture leverages the high lifting force of active systems with the energy-efficient torque profiles of passive elastic elements. The actuator will be a motor-driven novel helical actuator in conjunction with a linkage mechanism. The mechanism parameters can be tuned to provide optimal mechanical advantage between the motor and lifting force as a function of shoulder lifting angle. The spring component in parallel with the actuator will provide shoulder torque through a cam whose profile can also be optimized to tune the assistive load profile. One advantage of this system is that it is both backdrivable and also has spring components that can be relied on to partially support the lifting load in the case of power loss. Therefore, the proposed exoskeleton aims to combine improved lifting force of active exoskeletons with the energy capture and release ability of the elastic elements from passive exoskeletons.

## 2. Methodology

Safety and ergonomics are key factors in exoskeleton design. Device frame strength, controlled lifting force, and reliability are important safety considerations. This study covers the proof of concept for this exoskeleton device mechanism, and future work will consider frame strength, actuator torque, and controlled lifting force in the exoskeleton design. In that work, software and hardware limits to the max lifting torque and speed will be implemented among other safety systems. Weight, compactness, energy efficiency, and user friendliness are key ergonomic considerations. User friendliness will largely be determined by the control system developed in future work. The current design aims to maximize compactness, reduce the weight by utilizing lower torque actuators, and improve energy efficiency through the parallel spring-actuator system. Weight and energy efficiency will be improved through multiple design iterations.

The proposed hybrid exoskeleton aims to serve as a test-bed mechanism framework for different motion profiles. This stems from the substantial number of geometric mechanism parameters that can be varied during mechanism optimization. These parameters include linkage lengths, cam profile parameters, and helical actuator parameters. The helical actuator mechanism is included to offer more parameters to optimize, to achieve a multitude of mechanical advantage curves between the motor and the shoulder. Varied mechanism parameter sets will be optimized to achieve different mechanical advantage relationships which can be optimized for different tasks or applications. The helical actuator also can achieve large displacement ratios compared to other linear actuators, and this compactness advantage can be explored through mechanism optimization. The passive spring-cam system can produce a passive shoulder torque profile as a function of shoulder angle that can be tuned by optimizing the cam shape.

### 2.1. Helical Actuator Motivation

The majority of prismatic actuators used in robotics and machine design are either hydraulic cylinders, pneumatic cylinders, screw-based linear actuators, or belt-driven linear actuators. Some screw- and belt-driven actuators move a carriage between the fixed ends of the screw or belt. Examples of these include 3D printers and CNC machines. This research deals with actuators that change length during actuation and does not wish to make a comparison to fixed-end prismatic actuators since they often serve different applications.

Almost all hydraulic, pneumatic, and screw-based linear actuator cylinders have the same limitation that they cannot contract to more than twice their extended length. There are some multi-segmented telescoping versions of these three actuators, but they are uncommon and have other limitations. These limitations stem from subsequent segments having reduced diameters. This limits the bending moment that these actuators can withstand, and for hydraulic and pneumatic pistons, the cross-sectional area of the last segments is significantly less than that of the first segments which requires a much higher pressure to achieve the same linear force.

**Figure 1:**
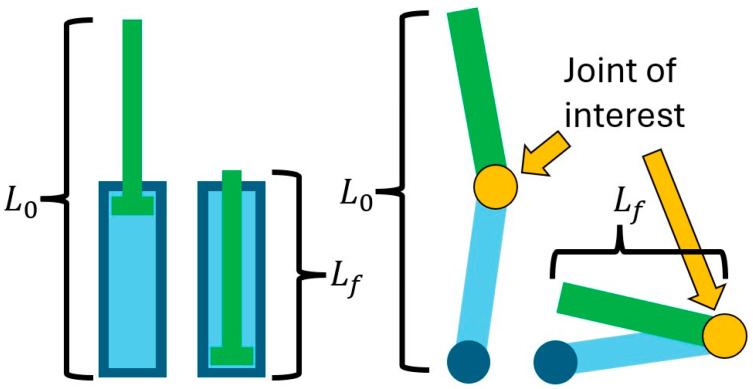
Prismatic and rotational actuators.

Most traditional robot mechanisms (again not including fixed-end gantries) that achieve linear motion will either use a two-segment piston/linear actuator or a combination of two rotational joints (Figure 1). In both of these mechanisms, the collapsed length of the mechanism (in its largest dimension) cannot be smaller than half of the extended length. This limits the reach of compact mechanisms.

There are other types of linear actuators that can contract to less than half of their extended length. These include polymer-based actuators like dielectric elastomers and liquid crystal elastomers [20,21,22,23]. These are limited by large activation voltage or large activation temperature, low response time, or low maximum applied force. Pneumatic muscles can also produce large relative displacements and output forces [24], but they cannot withstand bending loading and require external components to maintain rigidity in the extended state. Cable-based actuators [25] also require external rigid structures since wires cannot operate under bending or compression. Shape memory alloys can change their shape under applied voltage or heating [26], but this can be slow or produce small forces.

In addition to a limited extension-contraction ratio, the traditional rotation and piston-type linear actuators generally have a fixed mechanical advantage profile. This is generally preferred in many applications, but they cannot be used to optimize the mechanical advantage to different profiles over their range. The soft actuators have a variety of force, response time, and consistency limitations. The helical actuator allows for optimization of its mechanical advantage as well as an extension-contraction ratio of greater than 2:1 which opens up new optimization and compactness possibilities in exoskeleton mechanisms.

### 2.2. Exoskeleton Configuration

**Figure 2:**
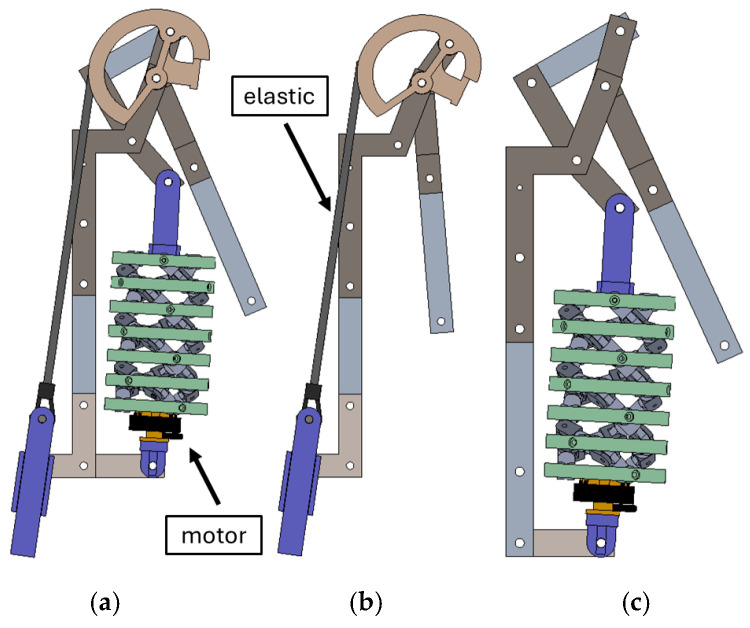
Exoskeleton spring and actuator. (**a**) Hybrid exoskeleton; (**b**) passive spring; (**c**) active helical actuator.

The helical actuator’s mechanism parameters along with the exoskeleton’s overall linkage parameters can be used to optimize the direct mechanical advantage profile between the actuator motor and the user’s shoulder and combined with optimizing the cam profile can even further optimize the mechanical lifting force dynamics. The entire mechanism is shown in Figure 2a with the motorized helical actuator and passive spring-cam system. Figure 2b and Figure 2c show the individual passive and active systems respectively.

The active subsystem will be evaluated based on the ability to optimize the mechanical advantage between the actuator motor rotation and the shoulder rotation. The passive subsystem will be evaluated based on the ability to optimize the cam profile to produce a desired lifting force as a function of shoulder angle.

This novel mechanism configuration aims to be configurable to have optimizable organic lifting dynamics. The long-term aim is to configure the mechanism to optimally match the human body’s lifting dynamics on a mechanical level in addition to a control software level.

## 3. Helical Actuator

**Figure 3:**
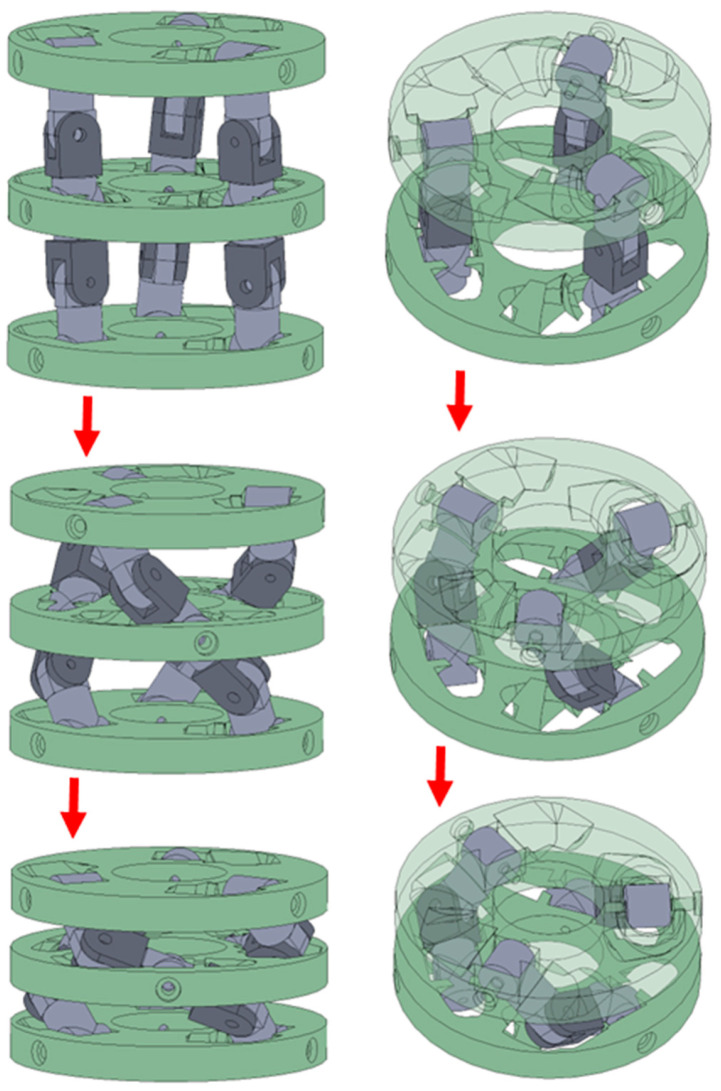
Helical actuator motion.

The helical actuator (Figure 3) is designed as a prismatic actuator capable of extending to more than twice its contracted length, making it well-suited for integration into compact mechanisms that require large displacements. In the literature, previous helical actuator versions have been used to actuate a bio-inspired inchworm robot [27,28], and two helical actuators have been configured in a concentric mechanism that allows for the control of the actuator displacement as well as rotation [29]. A key characteristic of the helical actuator is its nonlinear relationship between motor rotation input and linear displacement output. This relationship is continuous, invertible, and can be tuned through adjustments in linkage geometry and mechanism parameters. When combined with additional linkages, the actuator can be optimized to produce a mechanism with a highly customizable mechanical advantage profile. This capability will be leveraged in the design of the shoulder exoskeleton.

### 3.1. Helical Actuator Kinematics

**Figure 4:**
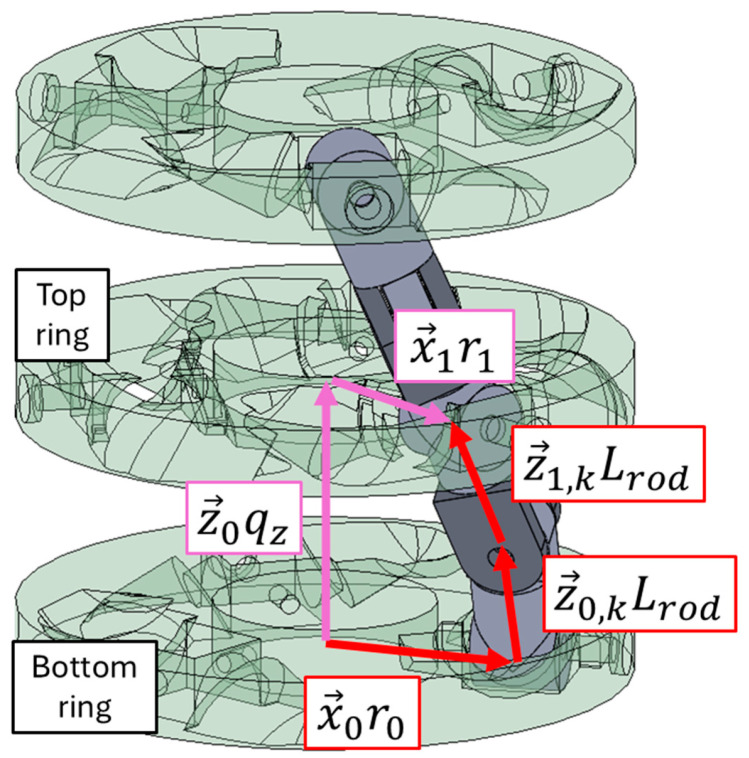
Helical actuator closed chain vectors.

The kinematics for the helical actuator are derived from 3D closed chain vector equations with the vectors shown in Figure 4. The helical actuator kinematics are solved as an inverse problem where the ring spacing $q_{z}$ is known, and the angle between adjacent rings $q_{\phi }$ is solved for.

The loop closure equations have two assumptions baked in: the rings stay parallel and concentric to each other. The equations only deal with the spacing between two adjacent rings and the three rod linkages that connect them. These are the bottom two rings and bottom three linkages shown in Figure 4. The first vector equation constrains two open chains. The first open chain (purple) travels from the origin of the bottom ring (frame $F_{0}$) up along the ${\vec{z}}_{0}$ vector by the known ring spacing distance $q_{z}$ to the origin of the top ring (frame $F_{1}$). The chain then travels along the top ring’s ${\vec{x}}_{1}$ axis by the pitch radius. The second chain travels along the bottom ring’s ${\vec{x}}_{0}$ axis by its pitch radius. It then travels up the rod linkages along the first two linkages’ $\vec{z}$ axes by the rod length. These two linkages are frames $F_{0,k}$ and $F_{1,k}$ respectively. The bottom ring’s origin is fixed in space, and it can only rotate along its $\vec{z}$ axis by $q_{\phi }$. The reference frames of rod $F_{0,k}$ are related to frame $F_{0}$ by the rotation of $\theta_{0,k}$ around ${\vec{x}}_{0}$. The subscript k ranges from 0 to 2 and represents each individual stock of rods. Rod frame $F_{1,k}$ is related to $F_{0,k}$ by rotating $\phi_{0,k}$ around ${\vec{y}}_{1,k}$, and rod $F_{2,k}$ is related to $F_{1,k}$ by rotating $\phi_{1,k}$ around ${\vec{z}}_{2,k}$. The first closed chain is

(1) $$e^{\hat{z}q_{\phi }}{\vec{x}}_{0}r_{0}+e^{\hat{z}q_{\phi }}e^{\hat{x}\theta_{0,k}}{\vec{z}}_{0,k}L_{rod}+e^{\hat{z}q_{\phi }}e^{\hat{x}\theta_{0,k}}e^{\hat{y}\phi_{0,k}}{\vec{z}}_{1,k}L_{rod}={\vec{z}}_{1}q_{z}+{\vec{x}}_{1}r_{1}$$

where the hat ($\hat{x}$) notation represents the skew-symmetric cross product matrix of a vector, and the exponential represents the matrix exponential to accomplish 3D rotations.

While the first closed chain constrains the position of the top ring, the second closed chain constrains its orientation. It relates the third rod ($F_{2,k}$) to the top ring ($F_{1}$) by the rotation of $\theta_{1,k}$ around ${\vec{x}}_{1}$. It is

(2) $$e^{\hat{z}q_{\phi }}e^{\hat{x}\theta_{0,k}}e^{\hat{y}\phi_{0,k}}e^{\hat{z}\phi_{1,k}}e^{-\hat{x}\theta_{1,k}}{\vec{x}}_{2,k}={\vec{x}}_{1}$$

It is noteworthy that in solving these equations, all unit vectors $\vec{x}$, $\vec{y}$, and $\vec{z}$ are coordinatized in their local frames and are ${\left[1\;0\;0\right]}^{T}$, ${\left[0\;1\;0\right]}^{T}$, and ${\left[0\;0\;1\right]}^{T}$ respectively. Also, the pitch radius of the bottom ring is a known constant $r_{0}$, but the pitch radius of the top ring $r_{1}$ is an unknown variable which changes slightly throughout the motion of the actuator. The top ring and every other ring like it are designed with a gap for the rod linkage $F_{2,k}$ to slide in the ${\vec{x}}_{2,k}$ direction.

These two 3D vector equations can be boiled down to the following six scalar equations:

(3) $$\begin{array}{c} L_{rod}\left(1+C_{\phi_{0,k}}\right)C_{\theta_{0,k}}=q_{z}\\ T_{q_{\phi }}r_{0}-S_{\theta_{0,k}}L_{rod}+T_{q_{\phi }}S_{\phi_{0,k}}L_{rod}-S_{\theta_{0,k}}C_{\phi_{0,k}}L_{rod}=0\\ S_{\phi_{0,k}}=T_{\theta_{0,k}}T_{\phi_{1,k}}\\ T_{q_{\phi }}C_{\phi_{0,k}}+C_{\theta_{0,k}}T_{\phi_{1,k}}+S_{\theta_{0,k}}S_{\phi_{0,k}}=0\\ C_{q_{\phi }}C_{\phi_{0,k}}C_{\phi_{1,k}}-S_{q_{\phi }}C_{\theta_{0,k}}S_{\phi_{1,k}}-S_{q_{\phi }}S_{\theta_{0,k}}S_{\phi_{0,k}}C_{\phi_{1,k}}=1\\ C_{q_{\phi }}r_{0}+\left(C_{q_{\phi }}S_{\phi_{0,k}}+S_{q_{\phi }}S_{\theta_{0,k}}\left(1+C_{\phi_{0,k}}\right)\right)L_{rod}=r_{1} \end{array}$$

where C, S, and T are cosine, sine, and tangent. These equations can be numerically solved for $\theta_{0,k}$, $\phi_{0,k}$, $\phi_{1,k}$, $\theta_{1,k}$, $q_{\phi }$, and $r_{1}$. Thus, the passive joint variables as well as the input angle can be calculated as a function of $q_{z}$.

**Figure 5:**
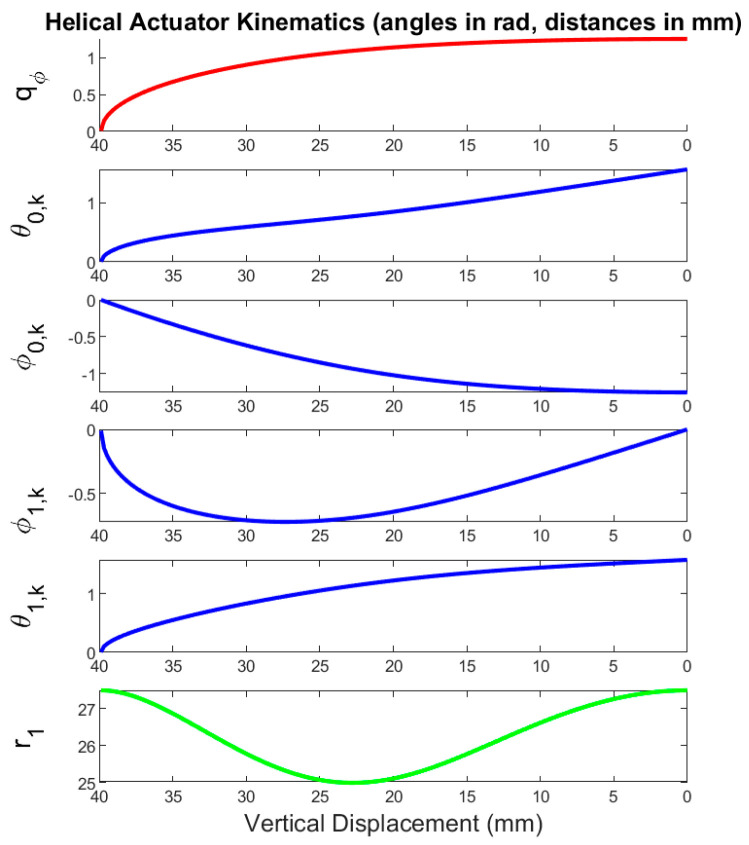
Helical actuator kinematics.

The plots for the helical actuator joints are shown as a function of actuator displacement in Figure 5. It is interesting that most of the variables have a very steep slope at the maximum displacement (~40 mm), since this is the state at which the change in displacement is most sensitive to input rotation.

### 3.2. Helical Actuator Statics

**Figure 6:**
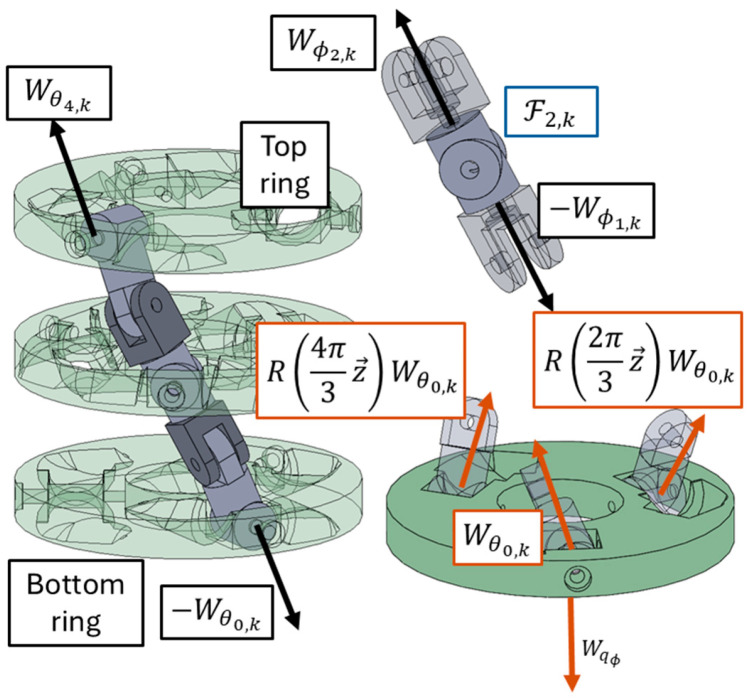
Helical actuator free body diagrams.

The goal of calculating the static forces in the helical actuator as a function of its displacement is to determine the relationship between the input motor torque and the output linear force. The reaction loads between different bodies are drawn in the free body diagrams (FBDs) in Figure 6. These loads are drawn as wrenches which are 6x1 vectors of force and moment: $W={\left[\begin{array}{cc} F^{T} & M^{T} \end{array}\right]}^{T}$.

The sum of forces and moments exerted on the bottom ring ($F_{0}$) at its origin is

(4) $$0=-W_{\phi_{z}}+\left[\begin{array}{cc} I_{3x3} & 0_{3x3}\\ \hat{x}r_{0} & I_{3x3} \end{array}\right]W_{\theta_{0,k}}+\left[\begin{array}{cc} e^{\frac{2\pi }{3}\hat{z}} & 0_{3x3}\\ \hat{e^{\frac{2\pi }{3}\hat{z}}\vec{x}r_{0}} & e^{\frac{2\pi }{3}\hat{z}} \end{array}\right]W_{\theta_{0,k}}+\left[\begin{array}{cc} e^{\frac{4\pi }{3}\hat{z}} & 0_{3x3}\\ \hat{e^{\frac{4\pi }{3}\hat{z}}\vec{x}r_{0}} & e^{\frac{4\pi }{3}\hat{z}} \end{array}\right]W_{\theta_{0,k}}$$

where the force between each stalk of rods and the rings is assumed to be radially symmetric, and $W_{\phi_{z}}$ is the reaction between the ring and its motor shaft. This equation reduces to

(5) $$W_{\phi_{z}}=3{\left[0\;0\;F_{\theta_{0,k},z}\;0\;0\;\left(M_{\theta_{0,k},z}+F_{\theta_{0,k},y}r_{0}\right)\right]}^{T}$$

where $F_{\theta_{0},z}$ is the z component of $F_{\theta_{0}}$, and likewise for $M_{\theta_{0},z}$ and $F_{\theta_{0},y}$. This result makes sense since due to the rotational symmetry assumption, there would be no net force or moment in the x or y directions on the ring.

The force between each of the rod linkages is the same since each linkage is only touching one other body at each of its ends. It is assumed that the rod $F_{2,k}$ that slides relative to the middle ring does not exert any force on that ring. The critical equation for solving these statics is the sum of moments of rod $F_{2,k}$ about its origin

(6) $$0_{3x1}=\hat{\left(\vec{z}L_{2,k}\right)}F_{\phi_{1,k}}$$

which produces $0=F_{\phi_{1,k},x}=F_{\phi_{1,k},y}$ and thus

(7) $$F_{\theta_{0,k}}=e^{\hat{x}\theta_{0,k}}e^{\hat{y}\phi_{0,k}}e^{\hat{z}\phi_{1,k}}\vec{z}F_{\phi_{1,k},z}={\left[\frac{T_{\phi_{0,k}}}{C_{\theta_{0,k}}}-T_{\theta_{0,k}}1\right]}^{T}F_{\theta_{0,k},z}$$

where $F_{\theta_{0,k},z}$ is the known axial load on the actuator. Next, the sum of the moments around $F_{0,k}$ and $F_{1,k}$ incorporating the zero-moment constraint of a passive pin joint are formulated as follows:

(8) $$0=-\left[\begin{array}{c} M_{\phi_{0,k},x}\\ 0\\ M_{\phi_{0,k},z} \end{array}\right]+\hat{\left(\vec{z}L_{1,k}\right)}e^{\hat{z}\phi_{1,k}}\vec{z}F_{\phi_{1,k},z}+\left[\begin{array}{c} M_{\phi_{1,k},x}\\ M_{\phi_{1,k},y}\\ 0 \end{array}\right]$$

which produces $M_{\phi_{1,k},y}=M_{\phi_{0,k},z}=0$. Then

(9) $$0=-\left[\begin{array}{c} 0\\ M_{\theta_{0,k},y}\\ M_{\theta_{0,k},z} \end{array}\right]+\hat{\left(\vec{z}L_{rod}\right)}e^{\hat{y}\phi_{0,k}}e^{\hat{z}\phi_{1,k}}zF_{\phi_{1,k},z}+e^{\hat{y}\phi_{0}}\left[\begin{array}{c} M_{\phi_{0,k},x}\\ 0\\ 0 \end{array}\right]$$

which produces $M_{\theta_{0,k},y}=L_{rod}\frac{T_{\phi_{0,k}}}{C_{\theta_{0,k}}}F_{\theta_{0,k},z}$ and $M_{\phi_{0,k},x}=M_{\theta_{0,k},z}=0$. Thus $M_{\phi_{z},z}=-3r_{0}T_{\theta_{0,k}}F_{\theta_{0,k},z}$.

**Figure 7:**
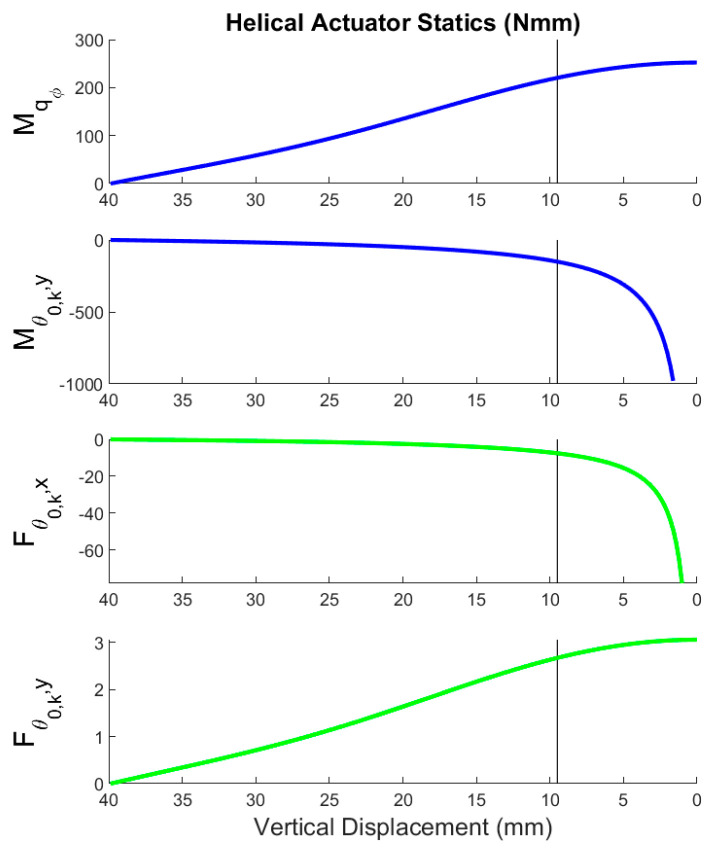
Helical actuator statics.(The vertical line indicates actuator displacement when fully folded.)

These forces are plotted as a function of displacement in Figure 7. The variables $F_{\theta_{0,k},x}$ and $F_{\theta_{0,k},y}$ both asymptote to negative infinity as the displacement goes to zero. The vertical lines represent the point at which adjacent rings contact each other and prevent further displacement, so the asymptotic behavior does not present an issue. This behavior makes intuitive sense using the analogy of a ladder sliding down a wall. The ladder’s vertical contact point on the wall represents the actuator’s vertical displacement, and the ladder’s horizontal contact point on the ground represents the ring rotation. The fully collapsed ladder is similar to the actuator fully collapsed. At that state, the horizontal force required to lift the ladder would asymptote to infinity.

## 4. Shoulder Exoskeleton

**Figure 8:**
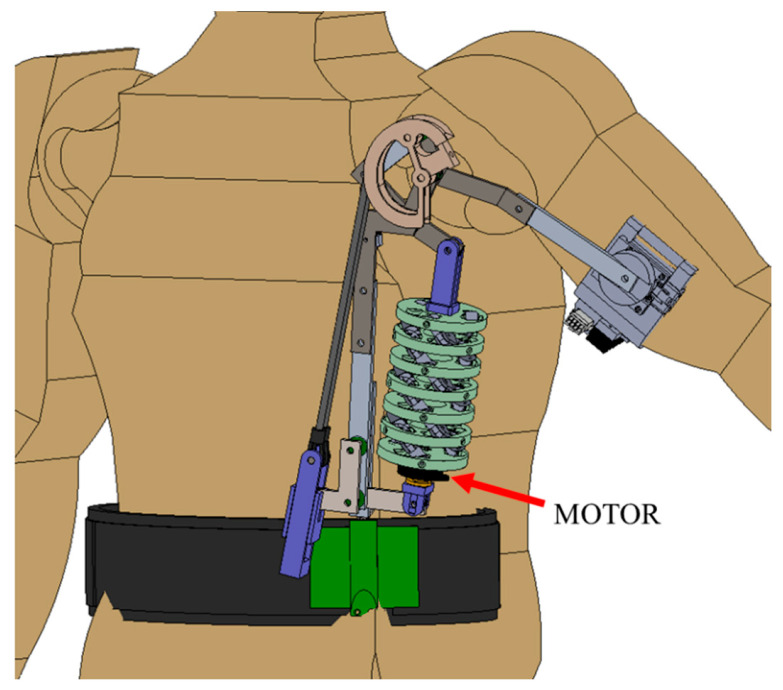
Hybrid exoskeleton.

The goal of this exoskeleton (Figure 8) is to provide assistive vertical force to the forearm when the user is performing lifting tasks. Due to Newton’s laws, in order for the exoskeleton to push up on the forearm, it must exert a reaction force at another location. This reaction force is realized at a mount on the user’s hip (Figure 8). Thus, with the exoskeleton in operation, the user’s shoulder and torso will be subjected to less load than without the exoskeleton, but the user’s legs will be subjected to the same load. For this reason, along with motor torque, material strength, and safety considerations, this exoskeleton is not intended to enable the user to lift more weight than they could without it. It is instead intended for users to carry the same weight that they otherwise would be carrying, but with the exoskeleton, their lifting force is reduced, which will reduce muscle strain and fatigue. Theoretically, in the event of actuator failure, the user could catch and hold the weight without exceeding the limits of their arm strength.

The exoskeleton consists of the following two main systems: the spring system and the actuation system. These both act in parallel to exert lifting torque on the shoulder joint. There is a metal frame that is supported by the ball joint at the hip belt, and it supports the shoulder joint. The spring system consists of a bungee cord, a tensioning mechanism, and a cam. The bungee is wrapped around the cam, and the cam profile will be optimized to provide the best lifting force profile. The actuation system starts with the MyActuator servo motor at its base which rotates the base of a helical actuator. The actuator used in this study had a ring pitch diameter of 55 mm. The extension and contraction serve as a prismatic joint which in turn actuates a 4 bar linkage system at the top. Originally, the system did not include the linkage mechanism, and the helical actuator directly lifted the final bar that supports the forearm. The problem with that setup was that the bar could only sweep through a limited range, and the user could not put their arm either straight down or straight up. This would be prohibitively restrictive to the user. This 4 bar linkage system is similar to that on the end of a construction excavator which controls the bucket. The helical actuator for this exoskeleton is similar to the prismatic hydraulic cylinder on the excavator which has the same range of motion issue. The linkage system actually decreases the mechanical advantage from the helical actuator to the shoulder motion, but it does increase the range of motion of the shoulder bar.

The support for the upper arm contains four force-sensitive resistors (FSRs) that detect the contact force between the arm and the support on the arm’s top, bottom, front, and back. Each of the FSRs are contained inside of a block that houses the FSR and a pressure plate that distributes the contact force on the arm on the one side and pushes against the FSR on the other side. Two of these sensor blocks are mounted rigidly to the support frame, and the other two blocks are held on by an elastic strap. The sensors will be used with an admittance control algorithm that uses elements of intension prediction to control the exoskeleton’s motor to either move smoothly with the user’s motion or lock the exoskeleton in place.

### 4.1. Shoulder Exoskeleton Kinematics

**Figure 9:**
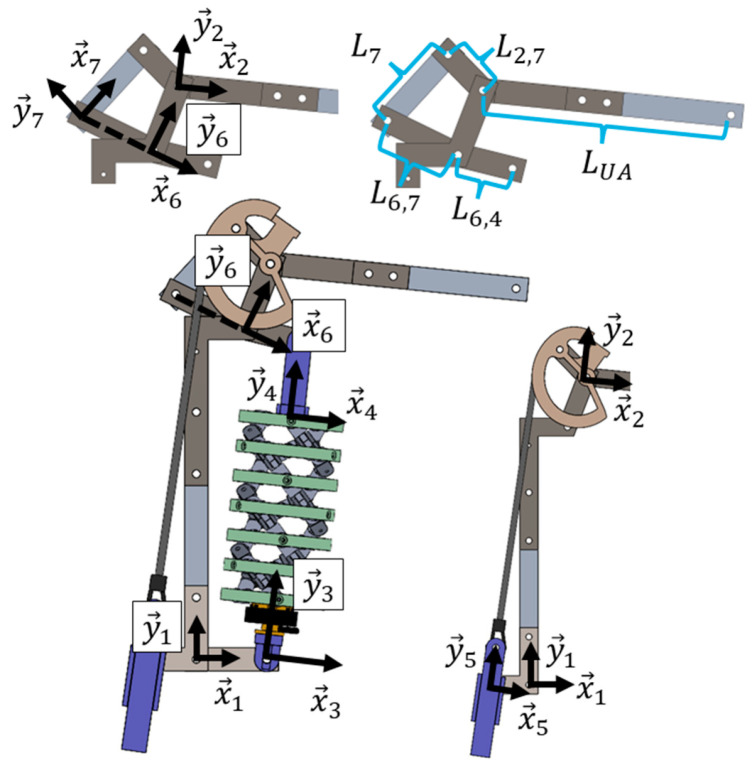
Linkage reference frames.

**Figure 10:**
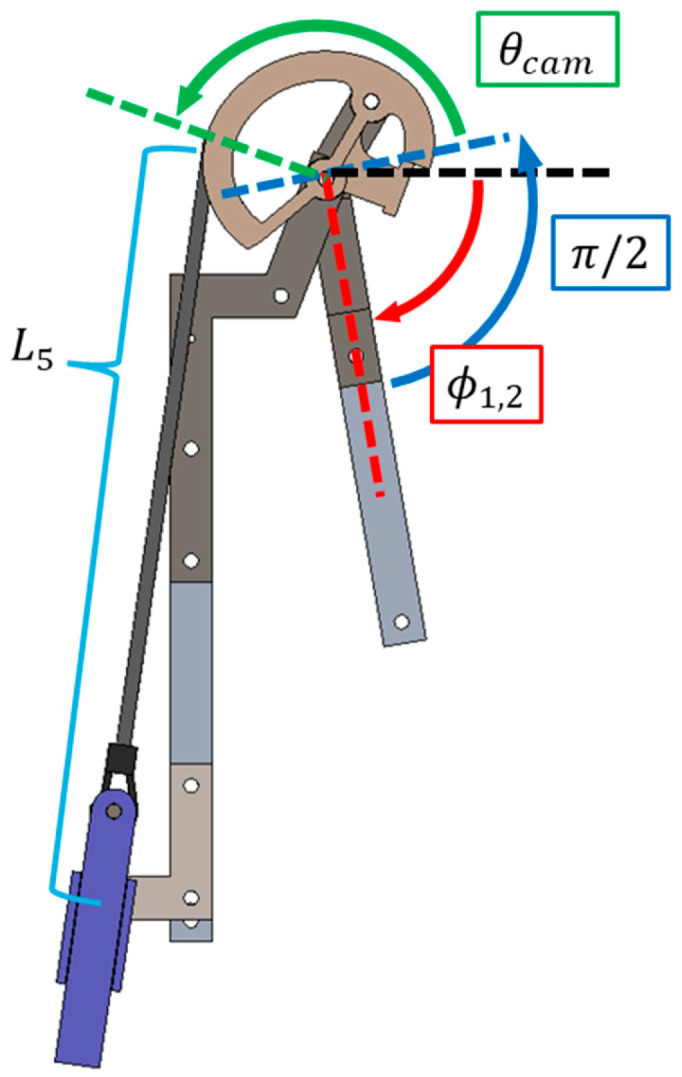
Cam angles.

The exoskeleton linkage inverse kinematics problem assumes a desired shoulder abduction angle and calculates the helical actuator displacement. This can then be used to solve the helical actuator inverse kinematics for the motor angle. The reference frames for each linkage are drawn in Figure 9. The linkage parameters of the exoskeleton include the vector from the origin of link 1 to that of link 2: ${\left[L_{1,2,X}\;L_{1,2,Y}\right]}^{T}$, the vector from the origin of link 6 to that of link 2: ${\left[L_{6,2,X}\;L_{6,2,Y}\right]}^{T}$, and the vector from the origin of link 1 to that of link 3: ${\left[L_{1,3}\;0\right]}^{T}$. The distance between the helical actuator pivot points that does not include the actuator displacement and is mostly taken up by the helical actuator brackets is $L_{4}$. Linkage lengths $L_{6,4}$, $L_{6,7}$, $L_{7}$, $L_{2,7}$, and $L_{UA}$ (UA → upper arm) are shown in Figure 10.

The first 2D loop closure equation circles the 4 bar linkage system that includes the helical actuator and its rocker $L_{6,4}$

(10) $$\left[\begin{array}{c} L_{1,2,X}\\ L_{1,2,Y} \end{array}\right]-\left[\begin{array}{c} L_{6,2,X}\\ L_{6,2,Y} \end{array}\right]+L_{6,4}\left[\begin{array}{c} C_{\theta_{6}+\phi_{1,6}}\\ S_{\theta_{6}+\phi_{1,6}} \end{array}\right]=\left[\begin{array}{c} L_{1,3}\\ 0 \end{array}\right]+\left(L_{4}+\left(n-1\right)q_{z}\right)\left[\begin{array}{c} -S_{\phi_{1,3}}\\ C_{\phi_{1,3}} \end{array}\right]$$

where n is the number of rings in the actuator, $\phi_{1,6}$ is the global angle of ${\vec{x}}_{6}$ relative to ${\vec{x}}_{1}$, $\phi_{1,3}$ is the global angle of ${\vec{x}}_{3}$ relative to ${\vec{x}}_{1}$, and $\theta_{6}$ is a constant parameter angle between ${\vec{x}}_{6}$ and the direction of the $L_{6,4}$ link (${\vec{x}}_{6}$ is in line with the $L_{6,7}$ link). The second loop closure equation encompasses the top four-bar linkage system of $L_{6,7}$, $L_{7}$, and $L_{2,7}$:

(11) $$\left[\begin{array}{c} L_{6,2,X}\\ L_{6,2,Y} \end{array}\right]-L_{2,7}\left[\begin{array}{c} C_{\phi_{1,2}-\theta_{2}}\\ S_{\phi_{1,2}-\theta_{2}} \end{array}\right]=-L_{6,7}\left[\begin{array}{c} C_{\phi_{1,6}}\\ S_{\phi_{1,6}} \end{array}\right]+L_{7}\left[\begin{array}{c} C_{\phi_{1,6}+\phi_{6,7}+\pi /2}\\ S_{\phi_{1,6}+\phi_{6,7}+\pi /2} \end{array}\right]$$

where $\phi_{1,2}$ is the angle between ${\vec{x}}_{2}$ and ${\vec{x}}_{1}$, $\phi_{6,7}$ is the angle between ${\vec{x}}_{7}$ and ${\vec{x}}_{6}$, and $\theta_{2}$ is the constant parameter angle between the arm bar $L_{2,7}$ and the negative ${\vec{x}}_{2}$ direction. Then, $\phi_{1,6}$ and $\phi_{6,7}$ can be solved as a function of $\phi_{1,2}$, and $q_{z}$ can be solved as a function of $\phi_{1,2}$.

The cam profile is defined to start π/2 rad counterclockwise from ${\vec{x}}_{2}$. The angular position of each point on the cam is $\theta_{cam}$ as shown in Figure 10. The radius $r(\theta_{cam}$) of the cam is defined as an n degree polynomial of $\theta_{cam}$. Thus

(12) $$r\left(\theta_{cam}\right)=\Sigma_{i=1}^{n}v_{i}\theta_{cam}^{i-1}$$

where $\left\{v_{i}|i=1,2,\ldots n\right\}$ is the set of polynomial constants. The cam kinematics problem is to find $\theta_{cam}$, $\phi_{5}$ (angle between ${\vec{x}}_{5}$ and ${\vec{x}}_{1}$), and $L_{5}$ (distance from origin 5 to where the elastic meets the cam as a function of $\phi_{1,2}$).

The cam kinematics is solved using the loop closure equation and by constraining the slope of the cam-elastic contact point to be the same as that of the elastic.

(13) $${\left({\vec{p}}_{1,5}\right)}_{1}+e^{\hat{z}\theta_{5}}yL_{5}={\left({\vec{p}}_{1,2}\right)}_{1}+e^{\hat{z}\left(\theta_{cam}+\theta_{1,2}+\frac{\pi }{2}\right)}\vec{x}r\left(\theta_{cam}\right)$$

(14) $$-\tan\left(\phi_{5}+\frac{\pi }{2}\right)=\frac{\partial p_{cam,y}}{\partial \theta_{cam}}/\frac{\partial p_{cam,x}}{\partial \theta_{cam}}$$

where ${\left({\vec{p}}_{1,5}\right)}_{1}={\left[-L_{5}\;0\right]}^{T}$, ${\left({\vec{p}}_{1,2}\right)}_{1}={\left[L_{1,2,X}\;L_{1,2,Y}\right]}^{T}$, and

(15) $$\frac{\partial p_{cam}}{\partial \theta_{cam}}=\left(\frac{\partial r\left(\theta_{cam}\right)}{\partial \theta_{cam}}+r\left(\theta_{cam}\right)\hat{z}\right)\left[\begin{array}{c} C_{\theta_{cam}+\theta_{1,2}+\frac{\pi }{2}}\\ S_{\theta_{cam}+\theta_{1,2}+\frac{\pi }{2}} \end{array}\right]$$

(16) $$\frac{\partial r\left(\theta_{cam}\right)}{\partial \theta_{cam}}=\Sigma_{i=1}^{n}\left(i-1\right)v_{i}\theta_{cam}^{i-2}$$

These equations can be solved numerically for $\theta_{cam}$, $\phi_{5}$, and $L_{5}$. The stretched length of the elastic along the cam is

(17) $$L_{cam}=\int_{0}^{\theta_{cam}}\sqrt{{\frac{\partial p}{\partial \theta_{cam}}}^{T}\frac{\partial p}{\partial \theta_{cam}}}d\theta_{cam}$$

**Figure 11:**
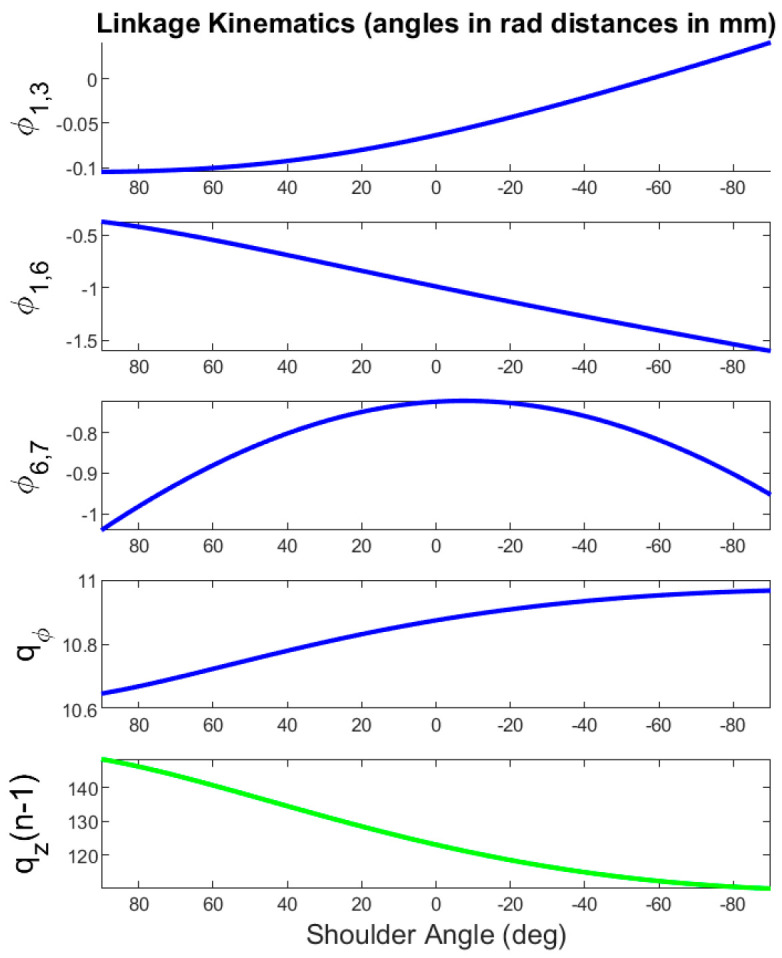
Linkage kinematics.

The linkage kinematics for a sample set of linkage parameters is shown in Figure 11. These are shown as a function of shoulder abduction angle where 90° is the arm pointing straight up, and −90° is the arm pointing straight down. These curves are sensitive to changes in the linkage parameters, and the parameters can be optimized to produce desirable curve shapes.

**Figure 12:**
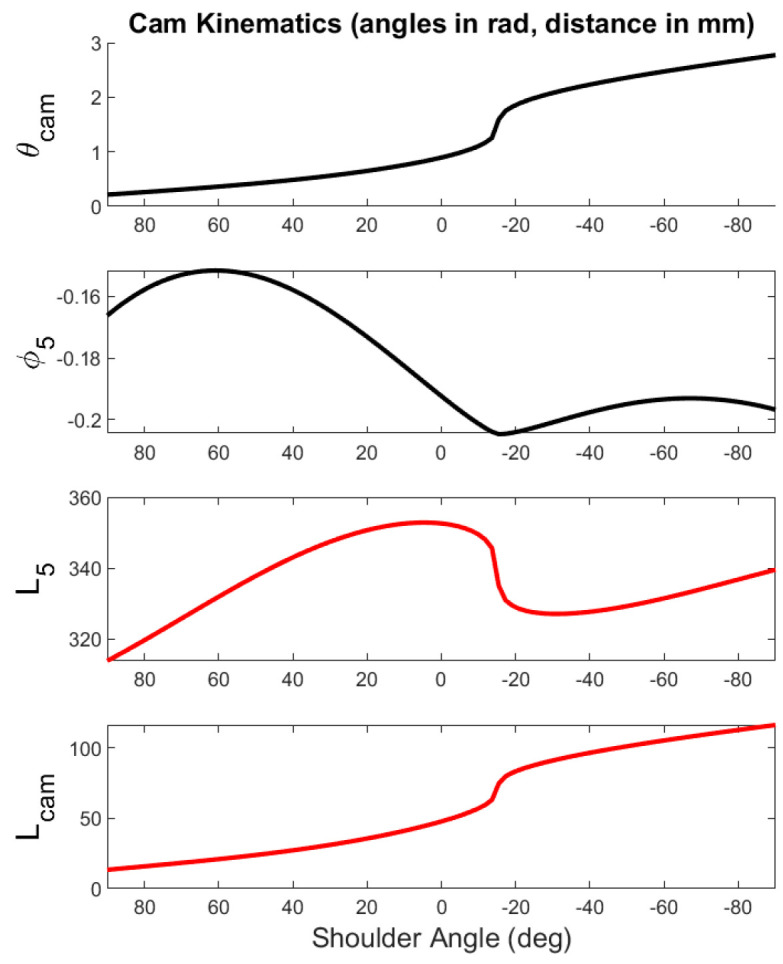
Cam kinematics.

The cam kinematics are shown in Figure 12. The angle $\theta_{cam}$ is the angle from the elastic contact point at which the elastic loses contact with the cam. This variable along with $\phi_{5}$, $L_{5}$, and $L_{cam}$ are sensitive to the cam profile parameters as well as the height and horizontal displacement between the elastic mount and the joint between links 1 and 2.

### 4.2. Shoulder Exoskeleton Statics

**Figure 13:**
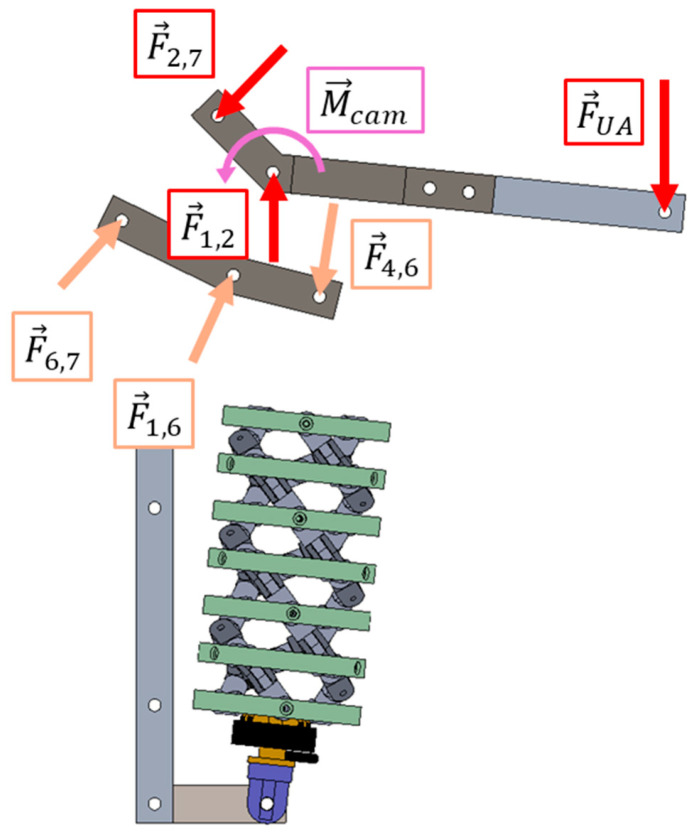
Exoskeleton free body diagram.

The statics calculations for the exoskeleton start with identifying an applied load on the upper arm bar from the user’s arm. The free body diagram for bodies 2 and 6 are shown in Figure 13. It includes reaction forces at the pin joints. It also includes the moment exerted on link 2 from the cam. A linkage that is pined on both sides (link 7 and the helical actuator) will only have reaction force at the pins, which is colinear with the line between the two pins. This comes from a sum of moments equation at any point about the link. Thus, the following perpendicular constraints are imposed: $0=x_{7}^{T}{\vec{F}}_{2,7}=x_{3}^{T}{\vec{F}}_{4,6}$.

The sum of moments around body 2 and 6 yields

(18) $$0=\left[\begin{array}{c} {{\vec{p}}_{2,UA}}^{T}\hat{z}\\ 0 \end{array}\right]{\vec{F}}_{UA}-\left[\begin{array}{c} C_{\phi_{1,5}-\left(\theta_{cam}+\theta_{1,2}+\frac{\pi }{2}\right)}\\ 0 \end{array}\right]r_{\theta_{cam}}\left(L_{preload}+L_{5}+L_{cam}\right)k_{bungee}+\left[\begin{array}{c} {{\vec{p}}_{2,2,7}}^{T}\hat{z}\\ {\vec{x}}_{7}^{T} \end{array}\right]{\vec{F}}_{2,7}$$

(19) $$0=\left[\begin{array}{c} {{\vec{p}}_{6,7}}^{T}\hat{z}\\ 0 \end{array}\right]\left(-{\vec{F}}_{2,7}\right)+\left[\begin{array}{c} {{\vec{p}}_{6,4}}^{T}\hat{z}\\ {\vec{x}}_{3}^{T} \end{array}\right]{\vec{F}}_{4,6}$$

where $L_{preload}$ is the preloaded distance that the elastic was stretched, and the various $\vec{p}$ vectors are the positions of the various forces relative to the origin of body 2 or 6 as appropriate. Equation (18) can be solved for ${\vec{F}}_{2,7}$ and then Equation (19) can be solved for ${\vec{F}}_{4,6}.$ Sum of the forces’ formulations can be used to calculate ${\vec{F}}_{1,2}$ and ${\vec{F}}_{1.6}$.

**Figure 14:**
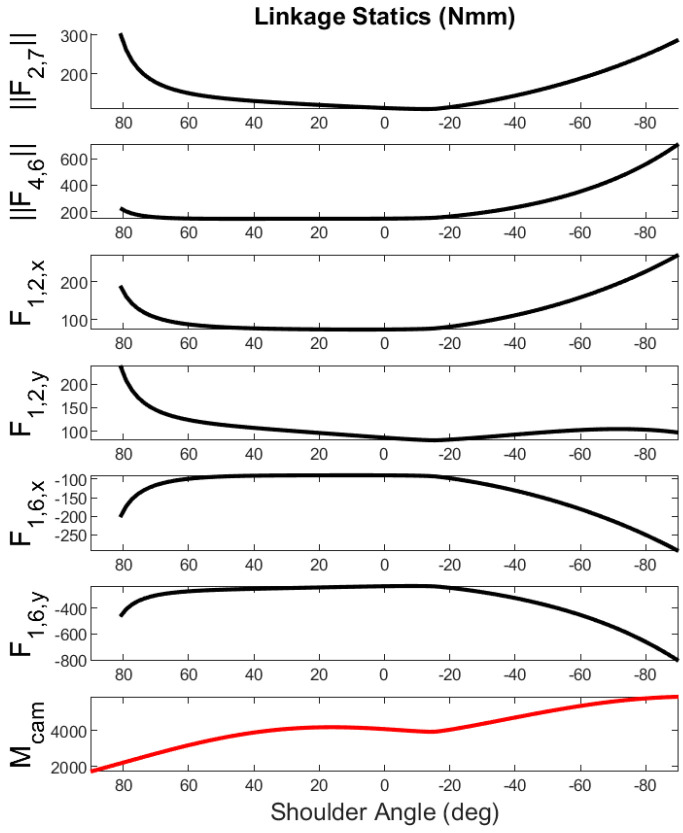
Linkage statics.

The reaction forces are shown in Figure 14, and these load profiles are sensitive to the input load between the arm and exoskeleton as well as the linkage parameters. The cam moment is also sensitive to the cam profile. In future work, the cam profile will be optimized to tune the cam lifting torque.

## 5. Prototyping

### 5.1. 3D Printing Parts

**Figure 15:**
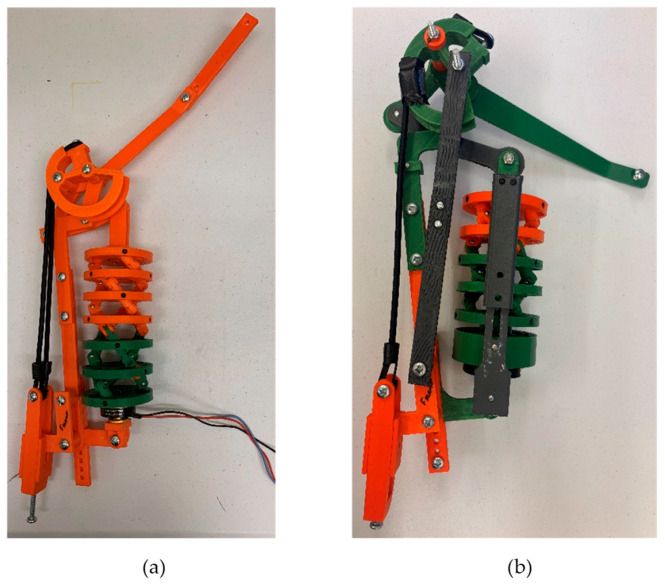
Exoskeleton prototype: (**a**) initial design; (**b**) new design with reinforcement frames.

Work has been started to 3D print a proof-of-concept prototype of the exoskeleton (Figure 15). This prototype will be used to continue developing the electrical system for exoskeleton. The control system was initially validated on a dummy, and once the simulation is fully robust, a metal-framed prototype will be fabricated to use for human testing.

### 5.2. Electrical System

The motor to drive the helical actuator must have fine position control, sufficient torque and precise position sensor feedback. Stepper motors can achieve precise feedforward position control, but most do not include position sensors for feedback control. Adding these sensors would be difficult while maintaining mechanism compactness. Brushed motors cannot provide precise low speed position control with high torque. Brushless DC (BLDC) motors can be selected with built-in position sensors, motor drivers, and gearboxes.

**Figure 16:**
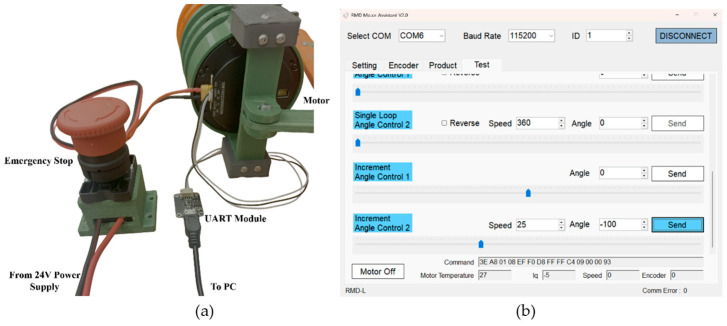
Electrical system for shoulder exoskeleton: (**a**) motor; (**b**) control interface.

A BLDC motor with integrated position sensor and position control loop was selected due to its compactness and control features. This was the MyActuator RMD-X6-P6-7 DRC version with CAN communication actuator powered by a 24V power supply with an emergency stop button in the main power line. It uses a universal asynchronous receiver-transmitter (UART) module to connect the motor via USB input into a computer as shown in Figure 16a. The MyActuator V2.0 software provides an interface to control the basic movement of the motor by adjusting angle and speed, then sending the command through a serial protocol as shown in Figure 16b. The angle of the motor was incremented; then, the arm lifting was observed. This communication protocol was selected as the easiest to implement quickly. In future work, an onboard controller will be used instead of the desktop computer, and other communication protocols and configurations will be investigated.

There is a limit switch that is mounted on the vertical frame of the exoskeleton that is pressed when the actuator extends to its limit. In the future, this motor can connect to a microcontroller through a CAN bus protocol using an MCP 2515 or another CAN controller instead of the UART module. This will allow for input processing from the four force sensitive resistors mounted on the arm cuff, as well as the limit switch.

## 6. Results

### 6.1. Kinematic Optimization

**Figure 17:**
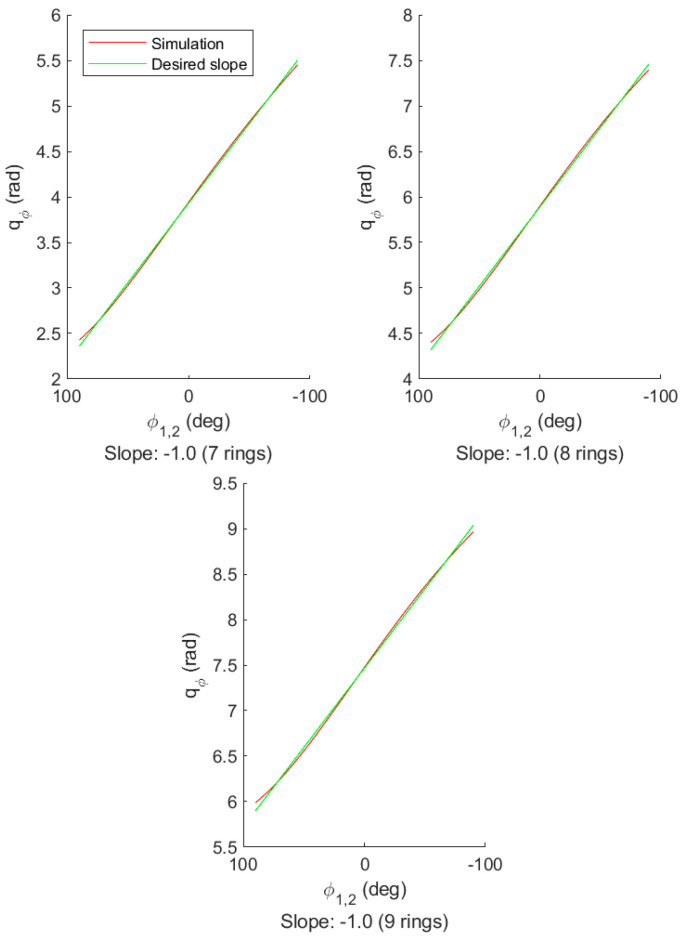
Mechanical advantage varying ring number.

**Figure 18:**
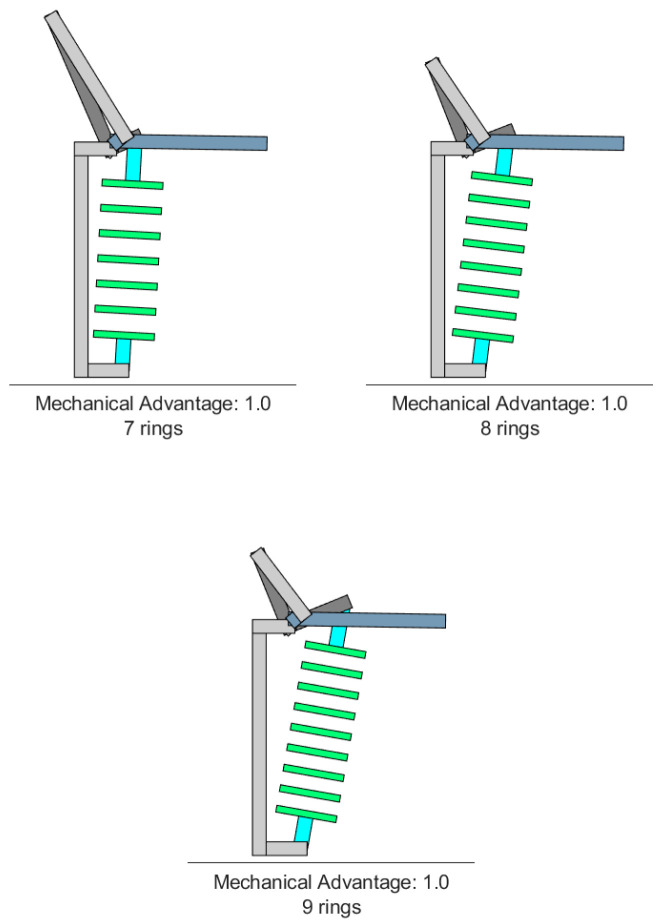
Exoskeleton parameters varying ring number.

The exoskeleton mechanism was optimized to achieve a desirable mechanical advantage. The objective function is to achieve the desired ratio between the input motor rotation of the actuator and the output shoulder rotation. There were 10 optimization design parameters which included eight linkage lengths and two linkage angles. We only impose the design variable bound constraints in the optimization formulation. The optimization problem was solved by Matlab R2026a fmincon function using interior-point method. Figure 17 shows three trials of the optimizer which linearized the motor rotation relationship with the shoulder rotation. A different number of actuator rings were used in each trial, and it is notable that they all achieved the same desired slope between shoulder rotation angle and motor rotation angle. The desired slope is shown as a green line, and the actual curve is red. They overlap very closely. The only difference between the trials is that they have an angular offset from each other, which is easy to account for in the motor control. The exoskeleton designs with the corresponding optimized parameters are shown in Figure 18. The spacing between the origins of F_6 and F_2 seemed to be minimized, and the main varying parameters are the rocker for the helical actuator and the crank and coupler for the arm linkage.

**Figure 19:**
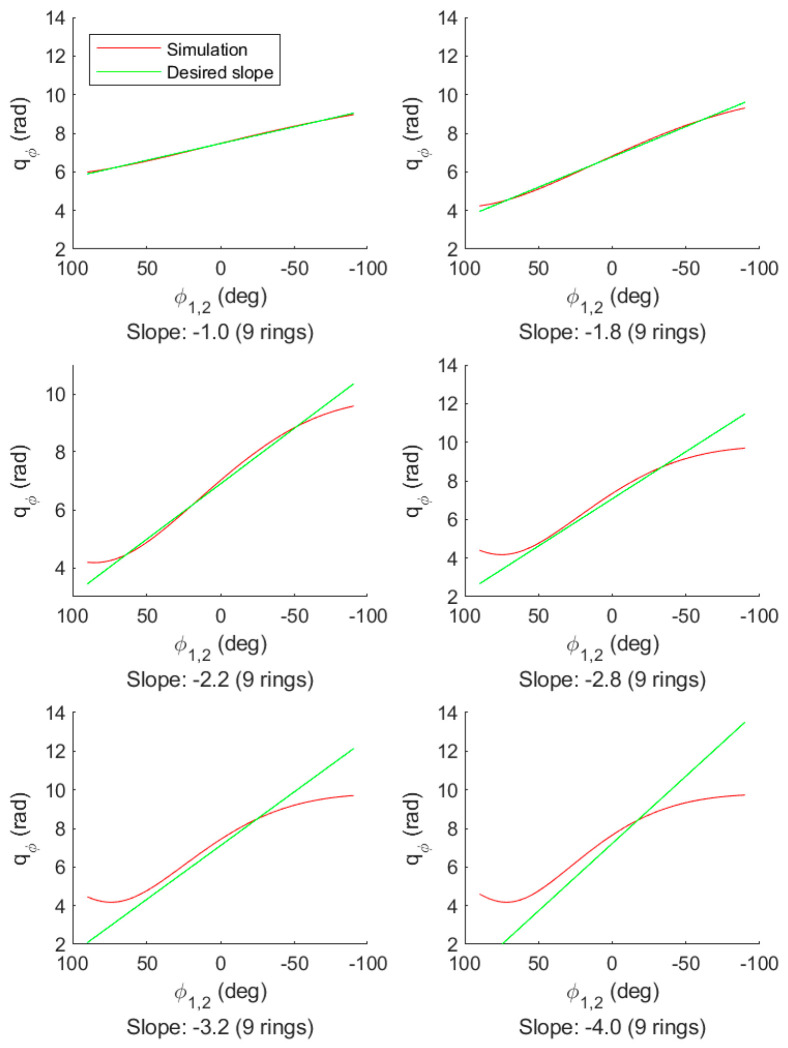
Mechanical advantage varying ring-desired slope.

**Figure 20:**
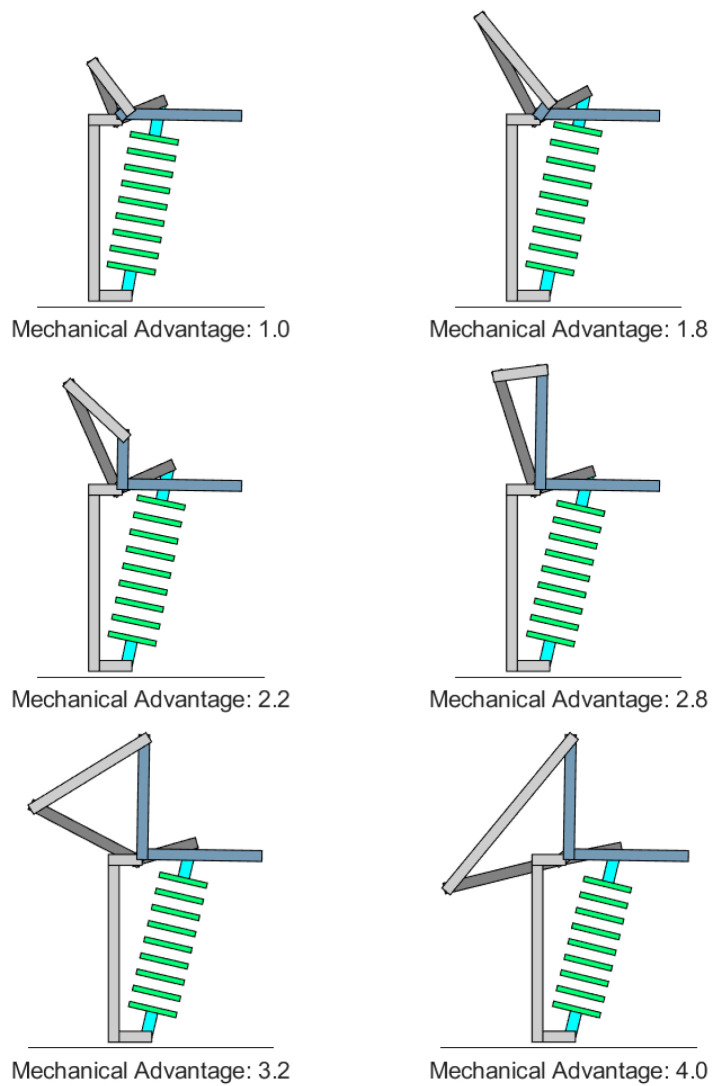
Exoskeleton parameters varying ring-desired slope.

The next set of trials kept the same ring number and varied the desired mechanical advantage slope between the shoulder angle and motor angle. The calculated relationship compared to the desired slope is shown in Figure 19, and the realized exoskeleton parameters are shown in Figure 20. The optimizer struggled to linearize the larger mechanical advantage ratios. This is probably because many of the linkage parameters had reached one of their upper or lower limits and the optimizer had fewer effective parameters to vary. Many of these solutions are not physically practical, and in future work, the joint limits and other constraints will be imposed to only allow for practical parameter sets. It will also utilize the statics calculations to ensure that the pins and linkages do not exceed their material limits.

This optimization produced some designs that were infeasible to implement due to the unwieldly upper linkages. The optimization process will be improved in future work. The results do demonstrate the ability for the combined helical actuator and linkage mechanism to be tuned to specific mechanical advantage curves. The solved configurations did not end up utilizing the full extension-contraction range of the helical actuator but did utilize its non-linear mechanical advantage and parameter tunability. The cam force optimization was not completed in this work. It will be completed in future work.

### 6.2. Experimental Results

**Figure 21:**
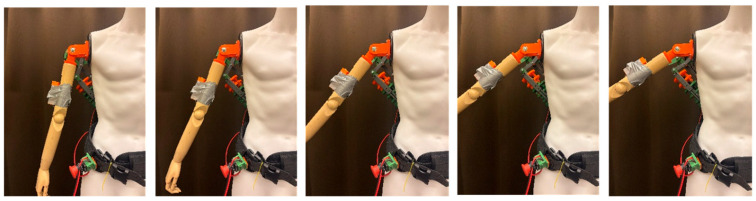
Snapshots of arm lifting by the hybrid shoulder exoskeleton.

The goal of this project is to demonstrate the usefulness of the helical actuator for the mechanism of a hybrid shoulder exoskeleton on a dummy. The motor and elastic band successfully raise the arm sequentially as shown in Figure 21. Initial results have been achieved, which will serve as a baseline for further dynamics simulations and more nuanced optimization goals. In future work, further iterations of the design will be load bearing while maintaining ergonomics and low weight.

## 7. Discussion and Conclusions

In this study, we designed and prototyped a hybrid shoulder exoskeleton which was driven by an active servo motor and a passive elastic band. The hybrid exoskeleton has features of more safety and power efficiency. The helical actuator and linkage system’s kinematics and statics were derived, which can be used to further optimize the system performance. The kinematics of the shoulder exoskeleton between motor rotation angle and shoulder rotation angle were optimized to achieve the desired slope. The servo motor and control were implemented in the system. The initial test was achieved successfully on a dummy. The results demonstrated the usability of the proposed hybrid shoulder exoskeleton. There are some limitations of the proposed design as follows: first, the optimization only considers the kinematics of the design, and statics and material strength limits are not considered; secondly, only functional validation is conducted; and third, the current device mass, mass distribution, and ergonomic acceptability are design constraints and future evaluation criteria need to be tested for real world ergonomics and long-term usability. In the current optimization formulation, only mechanism kinematics were optimized, and static-based constraints are not considered. This limits the practical design space. For future optimization formulation with statics, we will consider additional constraints on actuator torque, linkage frame material strength, exoskeleton lifting force, and human shoulder joint torque. We will integrate human-in-the-loop with the shoulder exoskeleton design. In addition, the actuator torque will be optimized as additional design variable along with the linkage dimensions to provide the user-appropriate assistive lifting force for different external load and human lifting motion. In the future, we will further develop advanced motor control systems, optimize the actuator and linkage dimensions to have better performance on assistive joint torque, and conduct human subject lifting experiments [30]. In addition, different materials will be used to manufacture the proposed exoskeletons [31,32].

## Data Availability

The original contributions presented in this study are included in the article. Further inquiries can be directed to the corresponding author.
